# Evidence of *Mycoplasma* spp. transmission by migratory wild geese

**DOI:** 10.1016/j.psj.2021.101526

**Published:** 2021-10-20

**Authors:** Anna Sawicka-Durkalec, Grzegorz Tomczyk, Olimpia Kursa, Tomasz Stenzel, Miklós Gyuranecz

**Affiliations:** ⁎ Department of Poultry Diseases, National Veterinary Research Institute, Puławy 24-100, Poland; † Department of Poultry Diseases, Faculty of Veterinary Medicine, University of Warmia and Mazury, Olsztyn 10-719, Poland; ‡ Institute for Veterinary Medical Research, Centre for Agricultural Research, Budapest 1143, Hungary

**Keywords:** *Mycoplasma* spp., phylogeny, waterfowl, wild geese

## Abstract

Mycoplasma infections have been found in different species of waterfowl worldwide. However, the question of how the pathogens have been transmitted and dispersed is still poorly understood. Samples collected from clinically healthy greater white-fronted geese (*Anser albifrons*) (N = 12), graylag geese (*Anser anser*) (N = 6), taiga bean geese (*Anser fabalis*) (N = 10), and barnacle geese (*Branta leucopsis*) (N = 1) were tested for *Mycoplasma* spp. All *Mycoplasma*-positive samples were specified by species-specific PCR for *Mycoplasma anserisalpingitidis* (formerly known as *Mycoplasma* sp. 1220), *M. anseris, M. anatis*, and *M. cloacale*. The presence of *Mycoplasma* spp. was confirmed in 22 of 29 sampled birds (75.9%). *Mycoplasma anserisalpingitidis* was the most frequently detected species (15 of 22, 68.2%). However, we did not detect any of the other *Mycoplasma* spp. typical for geese, among which are *M. anatis, M. anseris*, and *M. cloacale*. Phylogenetic analysis revealed that Polish sequences of *M. anserisalpingitidis* formed a distinct branch, along with 2 Hungarian isolates obtained from domestic geese. Eight of the samples identified as *Mycoplasma* spp.-positive were negative for the aforementioned *Mycoplasma* species. A phylogenetic tree constructed based on partial 16S rRNA gene analysis showed that *Mycoplasma* spp. sequences collected from Polish wild geese represent a distinct phylogenetic group with *Mycoplasma* sp. strain 2445 isolated from a domestic goose from Austria. The results of our study showed that wild geese could be a reservoir and vector of different species of the *Mycoplasma* genus that can cause significant economic losses in the domestic goose industry.

## INTRODUCTION

Wild waterfowl are reservoirs or vectors of many bacterial and viral diseases. Ordinarily, one pathogen can be spread to a broad range of bird species from different orders. However, mycoplasmas are known to be host-specific microorganisms. Usually, bird hosts are vectors of *Mycoplasma* species but infection does not develop clinical signs of disease in them (Gharaibeh and Hailat, 2011). However, an exception was discovered, where clinical signs were noticed in 2 peregrine falcons (*Falco peregrinus*) infected with *Mycoplasma gallisepticum* (**MG**), a common poultry pathogen. Those birds were kept in a rehabilitation center and carcasses of infected chickens were the possible source of infection (Poveda et al., 1990). One well-documented case of adaptation to a new host was the transmission of MG to wild passerines (Dhondt et al., 2014). Nevertheless, several previous reports described the spread of mycoplasmosis between wild and domestic birds of the same order. As a specific chicken and turkey pathogen, MG was detected in wild birds belonging to the *Galliformes* order such as lesser prairie chickens (*Tympanuchus pallidicinctus*) (Hagen et al., 2002) and wild turkeys (*Meleagris gallopavo*) (Luttrell et al., 1991; Fritz et al., 1992). However, clinical signs of disease were observed only in wild turkeys (Davidson et al., 1982). Respiratory disorders and sinusitis caused by MG infection were reported more frequently in game birds of the order *Galliformes* such as pheasants (Welchman et al., 2002; Bencina et al., 2003; Cookson and Shivaprasad, 2016), quails (Türkyılmaz et al., 2007), and partridges (Bradbury et al., 2001; Vitula et al., 2011). Literature data suggest that transmission of the pathogen was associated with contact of birds with infected commercial poultry (Hoffman et al., 1997; Bencina et al., 2003). However, phylogenetic analysis of MG isolates obtained from different avian species showed that the pathway of disease transmission could be bidirectional (Hochachka et al., 2013; Bekő et al., 2019).

Four mycoplasmas including *M. anseris, M. anatis, M. cloacale*, and *M. anserisalpingitidis*, formerly known as *Mycoplasma* sp. 1220, are specific to anserids. The presence of all these species was identified in both clinically healthy birds and birds with clinical signs of infection. The manifestation of clinical signs is usually associated with other environmental factors such as inadequate housing conditions or stress. Salpingitis, cloaca and phallus inflammation, airsacculitis, and embryo death are the most frequent symptoms in infected flocks of commercial waterfowl. In the literature, there are a few examples of *Mycoplasma* spp. isolation from healthy wild waterfowl. However, previous works have focused only on wild ducks. Poveda et al. (1990) were some of the first who confirmed the presence of *M. anatis* in northern shovelers (*Spatula clypeata*). The next cases from which *M. anatis* was isolated were described in American black ducks (*Anas rubripes*) and mallards (*Anas platyrhynchos*) (Goldberg et al., 1995). Another study conducted using the serological method proved high rates of *M. anatis* transmission among wild ducks (Samuel et al., 1996). Information on the occurrence of *Mycoplasma* spp. in wild geese is scarce. However, Gyuranecz et al. (2020) found that *M. anserisalpingitidis* isolated from Chinese swan geese (*Anser cygnoides*) showed high genetic similarity with the European isolates.

Our article describes the prevalence of *Mycoplasma* spp. in different species of healthy wild geese. *Mycoplasma*-positive samples were subjected to further molecular and phylogenetic characterization to investigate their epidemiological relationships. We consider if there is any common species of *Mycoplasma* spp. detected in wild geese, if there is a dominant species of wild goose that could be a reservoir of *Mycoplasma* spp., if migratory waterfowl could be a reservoir of *Mycoplasma* spp., and if there is evidence of transfer of *Mycoplasma* spp. between domestic and wild waterfowl.

## MATERIALS AND METHODS

### Sample Collection

Samples were taken from a total of 29 clinically healthy wild geese belonging to 4 species: the greater white-fronted goose (*Anser albifrons*; N = 12), graylag goose (*Anser anser*; N = 6), Taiga bean goose (*Anser fabalis*; N = 10), and barnacle goose (*Branta leucopsis*; N = 1). The geese were hunt prey between 2019 and 2020 in Warmińsko-Mazurskie voivodship, Poland (GPS 54.1006156, 21.0281888). All the birds were shot during regular hunts that were compliant with the legal regulations. No ethical committee permission was required as the swabs were collected postmortem. One sample (cloacal swab) was taken from a live barnacle goose that was captured by an ornithologist carrying out the Polish national avian influenza monitoring program in Gdynia, Pomorskie voivodship. The program is carried out by the laboratory of the Department of Poultry Diseases of the NRVI under the Council Directive 2005/94/EC of 20 December 2005 on Community measures for the control of avian influenza and repealing Directive 92/40/EEC85. No ethical permission was needed for such kind of study according to the Local Ethical Committee and Directive 2010/63/EU of the European Parliament and of the Council of 22 September 2010 on the protection of animals used for scientific purposes (Chapter I, article 1, p. 5 b, e, f). Oropharyngeal and cloacal swabs were taken from 24 birds while only cloacal swabs were taken from 4 taiga bean geese and 1 barnacle goose (Table 1). The samples were collected using swabs with a commercial transport system (ESwab Collection and Transport System, Copan Diagnostic, Murrieta, CA). The DNA was extracted directly from 200 µL of transport medium that was centrifuged at 20,000 × *g* for 60 s. The DNA was extracted using a QIAamp DNA Mini Kit (Qiagen, Hilden, Germany) following the manufacturer's recommendations and the extracted DNA was frozen immediately and stored at −20°C for further analyses.

**Table 1:** The number of individual birds tested for *Mycoplasma* spp. by polymerase chain reaction (PCR).

Species	*M.* spp.		*M. anseris*		*M. anatis*		*M. anserisalpingitidis*		*M. cloacale*	
	Positive/tested		Positive/tested		Positive/tested		Positive/tested		Positive/tested	
	Oropharyngeal	Cloacal	Oropharyngeal	Cloacal	Oropharyngeal	Cloacal	Oropharyngeal	Cloacal	Oropharyngeal	Cloacal
Greater white-fronted goose (*Anser albifrons*)	0/12	10/12	*nt*	0/10	*nt*	0/10	*nt*	8/10	*nt*	0/10
Graylag goose (*Anser anser*)	1/6	3/6	0/1	0/3	0/1	0/3	0/1	0/3	0/1	0/3
Taiga bean goose (*Anser fabalis*)	0/6	8/10	*nt*	0/8	*nt*	0/8	*nt*	6/8	*nt*	0/8
Barnacle goose (*Branta leucopsis*)	*nt*	1/1	*nt*	0/1	*nt*	0/1	*nt*	1/1	*nt*	0/1

*nt*, not tested.

### PCR Assay

A conventional PCR according to Lierz et al. (2007) with primers directed at the 16S rRNA gene was used with modifications as a screening method for detection of *Mycoplasma* spp. The PCR reaction mixture consisted of 2.5 μL of 10 × PCR buffer, 1 μL (10 μM) of deoxyadenosine triphosphate, deoxycytidine triphosphate, deoxyguanosine triphosphate, and deoxythymidine triphosphate, 0.5 μL of MgCl_2_ (1.5 mM), 1 μL of each primer (10 μM), 0.3 μL of OptiTaq polymerase (2 U, EURx, Gdańsk, Poland), 4 μL of Q solution (Qiagen, Hilden, Germany), 12.7 μL of water and 2 μL of the DNA sample. The following thermal cycling parameters were applied: 94°C for 4 min, followed by 40 cycles of 94°C for 30 s, 60.8°C for 1 min, and 68°C for 1 min, and a final extension of 68°C for 10 min. The reaction was performed in a T-Personal thermocycler (Biometra, Goettingen, Germany). The *Mycoplasma*-positive samples were tested for *M. anseris, M. anatis, M. cloacale*, and *M. anserisalpingitidis* using species-specific primers as specified by Grózner et al. (2019). All products amplified by PCR were separated by electrophoresis on a 2% agarose gel stained with ethidium bromide and subsequently visualized by UV transillumination.

### DNA Sequencing and Phylogenetic Analysis

Sequencing was performed on PCR products that produced strong bands following gel electrophoresis. Nucleotide sequences were determined by the Sanger method in a commercial laboratory (Genomed, Warsaw, Poland). Selected amplification products of the *rpoB* and 16S rRNA genes were sequenced in both directions with the forward and reverse amplification primers. Nucleotide sequences were compared and aligned with selected and publicly available sequences from the GenBank database. The sequences of the *rpoB* gene were deposited in the GenBank database under accession nos MW365715 to MW365727 and MW598190 to MW598191. These sequences of the 16S rRNA gene were also deposited in the GenBank database under accession nos MT374119, MT374249, MW355420, MW355421, MW355428, MW355429, MW355432, and MW355439.

To assess the phylogenetic relationship between *M. anserisalpingitidis*-positive samples, a phylogenetic tree was constructed for the *rpoB* gene (682 bp) using MEGA X 10.1 software (Kumar et al., 2018). The phylogenetic inference was based on the neighbor-joining genetic distance method using the maximum likelihood model. Bootstrap values were calculated based on 1,000 replicates and considered significant when >70.

A second phylogenetic tree was constructed for the 16S rRNA gene (851 bp) for other *Mycoplasma* spp.-positive and *M. anserisalpingitidis*-negative samples. Those sequences were compared with similar *Mycoplasma* species found in the GenBank database and *M. anatis* (accession no. CP030141.1), *M. anseris* (accession no. CP03140.1), and *M. cloacale* (accession no. CP030103) that were chosen as outgroup.

### Statistics

The statistical analysis was performed using R version 4.0.3 (R Core Team, 2020). The presence of differences in occurrence of *Mycoplasma* spp. between species of birds was verified using the chi-square test. Statistical significance was set at *P* ≤ 0.05.

## RESULTS

**Figure 1:**
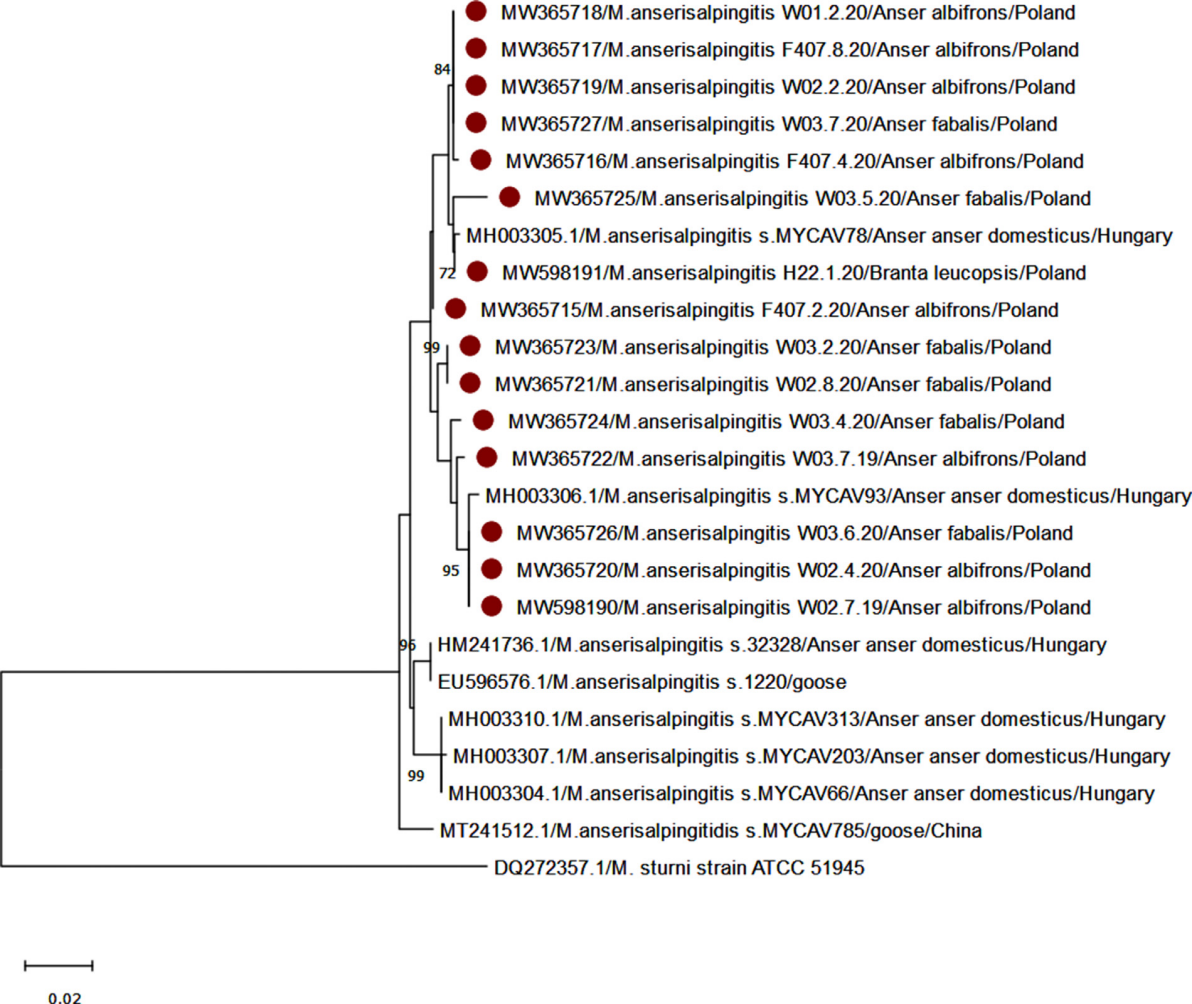
Phylogenetic analysis of partial sequences of the *rpoB* gene of *Mycoplasma anserisalpingitidis* detected in our study, highly similar isolates, and sequence of *M. sturni* used as the outgroup. The phylogenetic tree was constructed by the neighbor-joining method. Dark red circles indicate sequences obtained from this research.

The occurrence rates of *Mycoplasma* spp. in wild geese are presented in Table 1. The presence of *Mycoplasma* spp. was confirmed in 75.9% of tested wild geese, the bacterial DNA being detected more often in cloacal swab samples (75.8%) than in oropharyngeal ones (4.2%). We did not confirm significant differences in the occurrence of *Mycoplasma* spp. between species of geese (chi-square = 0.4518, *P* = 0.93). *Mycoplasma anserisalpingitidis* was found in 68.2% of samples from cloaca which were positive for bacteria of this genus. All *Mycoplasma*-positive samples were found to be negative for other species typical for geese such as *M. anseris, M. anatis*, and *M. cloacale*. The results of sequencing and phylogenetic analysis of the *M. anserisalpingitidis* isolates are shown in Figure 1. The similarity of our sequences to *M. anserisalpingitidis* isolates from Hungary and China ranged from 97 to 100% for the *rpoB* gene, on a fragment of which the phylogenetic tree was constructed, and it established 3 clades. All *M. anserisalpingitidis* sequences from our study formed a distinct branch along with 2 Hungarian isolates MYCAV78 and MYCAV93 (accession nos MH003305 and MH003306, respectively) that were obtained from domestic geese. The sequence similarities ranged from 99.6 to 100%.

**Figure 2:**
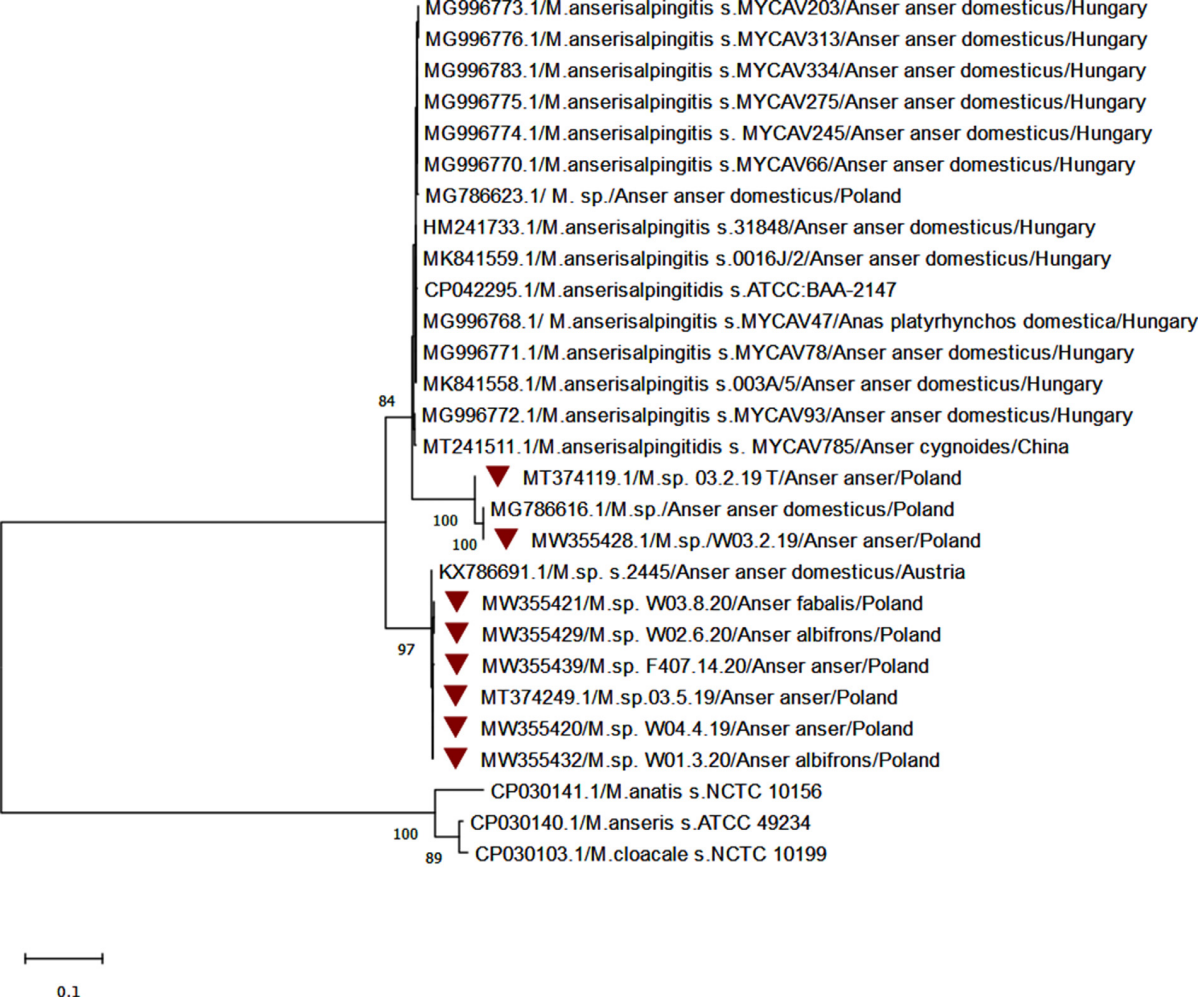
Phylogenetic analysis of partial sequences of the 16S rRNA gene of *Mycoplasma* spp. detected in our study, highly similar isolates, and sequences of *M. anatis, M. anseris*, and *M. cloacale* used as the outgroup. A phylogenetic tree was constructed by the neighbor-joining method. Numbers at nodes represent bootstrap confidence values (1,000 replications). Dark red triangles indicate sequences obtained from this research.

The phylogenetic tree for the 8 sequences based on 16S rRNA showed no strong consistent pattern for associations between host species or country of isolation (Figure 2). However, the sequences obtained in our study could be divided into 3 groups. Sequences in the first group clustered with Hungarian isolates from domestic geese and ducks and were particularly close to an isolate obtained from a swan goose from China (accession no. MT241511.1). Sequence similarities in this group were within the range of 99.4 to 100%. The second group contained sequences from tracheal and cloacal swabs collected from the same wild geese that were similar (99.5–100%) to a sequence of *Mycoplasma* spp. isolated from a domestic goose in Poland (accession no. MG786616.1). The last group contained *Mycoplasma* spp. sequences collected from different species of Polish wild geese (accession nos MT374249.1, MW355420.1, MW355421.1, MW355429.1, MW355432.1, and MW355439) and an Austrian isolate (accession no. KX786691.1) of *Mycoplasma* sp. strain 2445 obtained from a domestic goose. Sequence similarities in this group were within the range of 99.6 to 100%.

## DISCUSSION

The results obtained in our study show a high occurrence of *Mycoplasma* spp. in wild geese. Twenty-two of the 29 wild geese were positive by PCR analysis using *Mycoplasma* genus-specific primers. Fifteen of these 22 samples were positive for *M. anserisalpingitidis*. Our results were similar to those found in domestic waterfowl. Stipkovits and Szathmary (1996) reported finding *M. anserisalpingitidis* as the most frequent species. Volokhov et al. (2020) interpreted sequence analyses and suggested that the *rpoB* gene of *Mycoplasma anserisalpingitidis* could vary by approximately 4% between strains. It is also notable that analysis of the partial *rpoB* gene showed high similarity of our sequences to *M. anserisalpingitidis* isolates from distant geographical regions such as Hungary and China (Gyuranecz et al., 2020). The authors found a close relationship between the *M. anserisalpingitidis* MYCAV93 strain isolated from the inflamed phallus of a domestic goose in Hungary in 2011 and MYCAV785 strain isolated from a clinically healthy swan goose from China. Our findings confirmed the hypothesis of Grózner et al. (2021) that this *Mycoplasma* spp. could be transmitted by migratory birds. Knowledge of the occurrence of *Mycoplasma* spp. in wild waterfowl is limited (Astorga et al., 1994; Goldberg et al., 1995; Samuel et al., 1996); however, wild waterfowl are generally known to be reservoirs and vectors of many bacterial diseases (Elmberg et al., 2017).

We believe that wintering or breeding areas of wild waterfowl could be places of infection. Bairlein (2001) mentioned that migration connects different species of geese in the same area of breeding or wintering. After the breeding season, a large part of the population of wild birds migrates to the Netherlands for overwintering. Moreover, taiga bean geese and greater white-fronted geese also winter in large numbers in Hungary (BirdLife International, 2021a,b). This could be a possible explanation of why the same *Mycoplasma* spp. are found in Polish and Hungarian geese. Our study highlights the role of wild geese in transmission of *Mycoplasma* spp. between commercial waterfowl flocks. During migration, birds make stopovers and these sites could be potential places of direct contact between wild and commercial waterfowl and therefore of horizontal transmission of pathogens. It could be especially dangerous for breeder geese. Specific practice in the husbandry of breeding flocks of commercial geese which allow free access to pasture and water increases the risk of contact with wild geese and the transmission of pathogens. Additionally, Stipkovits et al. (1993, 1987) found that *M. anserisalpingitidis* and some *Mycoplasma* spp. strains could be transmitted vertically as well as horizontally. We hypothesize that the high occurrence of *Mycoplasma* spp. obtained in our study could be explained by the easy spread of *Mycoplasma* in the wild goose population. The high occurrence of *Mycoplasma* spp. and the absence of clinical symptoms in wild geese may suggest persistent infection (Rottem, 2003). The presence of *M. anserisalpingitidis* was also reported in clinically healthy flocks of commercial geese (Hinz et al., 1994). The development of clinical signs of mycoplasmosis in birds could be caused by inadequate environmental factors, the onset of egg production or other stressors (Stipkovits and Kempf, 1996; Kleven, 1998).

Eight of the *Mycoplasma* spp.-positive samples were negative in PCR assays targeting *M. anserisalpingitidis, M. anatis, M. anseris*, and *M. cloacale*. The phylogenetic analysis of those sequences showed a division of the tested samples into 2 groups. It is worth noting that the similarity between the groups of sequences in our study was below the 94% threshold for species discrimination using 16S rRNA gene sequences (Brown et al., 2007). It may suggest the detection of other unknown, novel species of *Mycoplasma*. It should additionally be emphasized that one such unknown species of Mycoplasma was also detected in Austrian domestic geese, which suggests that it could be transmitted from wild birds. The presence of unidentified *Mycoplasma* species was also reported in breeder geese in Germany (Behr et al., 1990) and the United States (Carnaccini et al., 2016). Moreover, some clinical signs such as phallic deformities may also have been caused by unidentified *Mycoplasma* species (Behr et al., 1990; Carnaccini et al., 2016). In previous studies conducted by Hinz et al. (1994), multiple *Mycoplasma* infections in geese were also reported. These findings mainly concerned the simultaneous isolation of mycoplasmas commonly known as typical for the goose, such as *M anserisalpingitidis, M. anatis, M. anseris*, and *M. cloacale*. Detection of the same *Mycoplasma* species in domestic and wild geese inspired us to further investigate the potential role of this microorganism in the pathology of commercial waterfowl.
